# Using structural bioinformatics to investigate the impact of non synonymous SNPs and disease mutations: scope and limitations

**DOI:** 10.1186/1471-2105-10-S8-S9

**Published:** 2009-08-27

**Authors:** Joke Reumers, Joost Schymkowitz, Fréderic Rousseau

**Affiliations:** 1 Switch Laboratory, VIB, Vrije Universiteit Brussel, Pleinlaan 2, 1050 Brussels, Belgium

## Abstract

**Background:**

Linking structural effects of mutations to functional outcomes is a major issue in structural bioinformatics, and many tools and studies have shown that specific structural properties such as stability and residue burial can be used to distinguish neutral variations and disease associated mutations.

**Results:**

We have investigated 39 structural properties on a set of SNPs and disease mutations from the Uniprot Knowledge Base that could be mapped on high quality crystal structures and show that none of these properties can be used as a sole classification criterion to separate the two data sets. Furthermore, we have reviewed the annotation process from mutation to result and identified the liabilities in each step.

**Conclusion:**

Although excellent annotation results of various research groups underline the great potential of using structural bioinformatics to investigate the mechanisms underlying disease, the interpretation of such annotations cannot always be extrapolated to proteome wide variation studies. Difficulties for large-scale studies can be found both on the technical level, i.e. the scarcity of data and the incompleteness of the structural tool suites, and on the conceptual level, i.e. the correct interpretation of the results in a cellular context.

## Background

The molecular phenotype of a coding non synonymous SNP or disease associated mutation describes the functional and structural properties of a protein that are affected by a single amino acid substitution [1]. In this study we want to address whether the concept of the *in silico *determined molecular phenotype can be employed for large-scale classification of SNPs and disease mutations. The attempt to classify a large set of mutations based on an incomplete molecular phenotype may seem naive at first glance, had it not been suggested that individual properties such as protein stability, the accessibility of the amino acid substitution site, and the location of variants in surface pockets are predictive determinants of the phenotypic effect of a variation [2-5]. A comparative study of protein stability predictors by Blundell and co-workers demonstrated that although protein stability changes caused by mutation can be relatively accurately estimated *in silico*, these predictions by themselves do not yield accuracy on large-scale classification between benign and disruptive mutations [6-8].

Furthermore, computational analyses rely heavily on the quality of the data under scrutiny and the computational methods used to evaluate these data. Before investigating 39 structural properties of proteins and amino acid substitutions for their predictive power regarding SNP classification, we have investigated what major liabilities are encountered when implementing an structural approach to SNP annotation and classification. The results are compared with those achieved by the best performers among the state-of-the-art tools.

## Results and discussion

In this study we have identified the common issues that are encountered when performing large-scale analyses of structural properties of human coding variation. The first issue concerns the availability of structural data for nsSNPs and disease mutations, while the second involves the availability of computational tools to predict structural properties. The last issue concerns the quality of classification: are the training and evaluation data sets used in the analyses sufficient to extrapolate results for larger studies, and do the properties used have sufficient predictive power to separate the two data sets?

### Structural coverage of human genetic variation

Despite structural genomics projects, the gap between sequence and structural information is still wide, and the coverage of variation data with structural data is estimated to be as low as 14% [5]. We have investigated the boundaries of structural coverage by varying the quality requirements on the structural model (Supplementary Figure S1A in Additional file 1), the sequence identity between query sequence and modelled structure (Figure S1B), the percentage of the wild type sequence covered by the structural model (Figure S1C), and the length of the alignment between query and target (Figure S1D). Circa 12% of all nsSNPs present in the Ensembl Variation Database (release 44) can be mapped on a structural model, in accordance with the estimate cited previously. However, this percentage is valid only when no restrictions regarding sequence identity, sequence coverage or structure quality are applied.

In Figure S1A we see that the number of SNPs covered by structural data drops after 40% sequence identity. Requirements on sequence identity sufficient for prediction are different for various methods. Yue & Moult [5] found a sequence identity of 40% sufficient for accurate prediction, while Chasman & Adams [2] obtained the best results with identities higher than 60%. However, these methods do not use full atomic detail to assess the structural properties of an amino acid substitution, and thus to do not require high sequence identity to be able to model the substitution. We use the FoldX force field to model amino acid variation on structural models, which uses an all-atom representation of the structure. Although this introduces high accuracy of stability estimation [7,9,10], it also requires high quality structural models. Our standard restrictions on building high-confidence structural models using the FoldX force field are X-ray structures with a resolution lower than 2.5 Å and sequence identity higher than 80%. Applying these restrictions to the Ensembl data results in a data set of 5416 nsSNPs (circa 4% of the data, Figure S1B).

Other factors in fluencing the structural coverage of SNPs is the length of the alignment and the percentage of coverage between the query sequence and the structural model. A realistic criterion for human proteins to apply would be to request the structural alignment to be about 100 amino acids long, or, for proteins shorter than 100 residues, to cover more than 80% of the query sequence. When this criteria are combined with the need for high quality structural data, we find that 8238 nsSNPs remain in the data set. A summary of the number of SNPs covered by high quality structural data, in combination with criteria regarding the reliability of the nsSNP data, is shown in Table 1. In this table we see that the application of stringent criteria will result in the structural mapping of very few nsSNP data.

**Table 1:** Summary of structural coverage of SNP data.

**Properties**	**# SNPs**	**% SNPs**
*nsSNPs covered by high quality structural data*		
No additional criteria	9877	7.4
Sequence coverage > 80 or alignment length > 100	8238	6.2
Sequence identity > 80	5416	4.1
Sequence coverage > 80 or alignment length > 100, and sequence identity > 80	5318	4.0
*Highly reliable nsSNPs covered by high quality structural data*		
Doublehit validation status, MAF > 0.01	680	0.51
Doublehit validation status, MAF > 0.01, sequence identity > 80	229	0.17
Doublehit validation status, MAF > 0.01, sequence coverage > 80 or alignment length > 100	446	0.33
Doublehit validation status, MAF > 0.01, sequence coverage > 80 or alignment length > 100, and sequence identity > 80	209	0.16

Several criteria resulting from the above analyses are applied to assess the structural coverage and reliability of that coverage of human SNPs in the Ensembl database, as well as the overlap of the structural coverage with quality parameters for the validation and frequency status of the polymorphism data.

### Predictability of structural properties

The second issue for a large-scale structural bioinformatics approach is the structural properties that are predictable with state of the art tools: how well can we describe the structural behaviour of a protein and its mutants? Previous structural studies have identified protein stability, aggregation and misfolding as determinants of correct functioning on the single protein level [8,11,12]. Mutations affecting the functional sites of a protein, such as DNA, ligand and protein interaction sites, are not considered within this scope, but the investigation of these sites will most certainly be of great importance to assess the impact of amino acid substitutions.

Tools have been developed that describe the structure and dynamics of a protein: stability, aggregation, amyloidosis, and folding. We have used computational methods that are capable of assessing the effects of a mutation on protein stability (FoldX), aggregation (Tango) and amyloidosis (Waltz). Although algorithms exist that can predict folding of small single domain proteins (e.g. Rosetta [13], FoldX [14], SimFold [15]), to date no computational method exists that can predict folding events on large multi-domain proteins, or that is applicable in genome wide studies.

Although we have limited ourselves to the analysis of structural features of single protein molecules, and have not investigated protein-protein interactions in this study, we have included an analysis of the binding of proteins to molecular chaperones, as it is directly related to correct folding of the protein. The high abundance of chaperones in the cell emphasises their crucial role in the protein quality control system [16], but this is not reflected in the availability of computational tools for chaperone binding. We have used the only available tool, the Hsp70 binding predictor Limbo [17], to assess chaperone binding variation caused by amino acid alteration.

### The predictive power of structural properties

Following the recommendations of Care *et al *[18], we have used the SwissProt annotated disease and polymorphism data (SwissProt Variation Index release 52) as the evaluation data for our analyses. Mapping of these variants on high quality structural models (X-ray structures with resolution ≤ 2.5 Å, sequence identity with the model above 80%) yielded a data set of 240 positive (disease-associated) mutations and 400 negative variations (neutral nsSNPs) in 98 proteins. To ensure that the analyses are comparable, we applied the sequence based predictors to the same small data set as the predictors that use 3D structures or structural models.

Before we evaluated the discriminative power of the individual structural parameters, we wanted to assess whether our data showed distinguishable patterns for three important parameters. The first two criteria, stability difference and the degree of burial of the mutation site, have previously been identified as providing information about the severity of a mutation [5,19]. The third criterion is difference in aggregation propensity, which has been cited as likely to be an important factor in disease susceptibility [12,20] but thus far has not been applied in a proteome wide mutation analysis.

**Figure 1:**
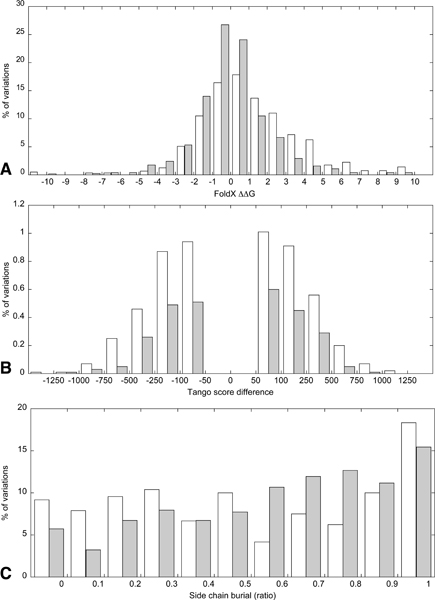
**Distributions for the major structural criteria in the disease and polymorphism datasets**. White = disease mutations, grey = polymorphisms. **A**. Stability difference as calculated by the FoldX force field (in kcal.mol^-1^). **B**. Difference in aggregation propensity as calculated by the Tango algorithm. Values close to neutral changes (in the range [-50, 50]) are left out for display purposes. **C**. Distribution of degree of burial of the amino acid substitution site.

Figure 1 shows the distributions for the stability differences (A) and differences in aggregation propensity (B) between wild type and variant proteins, and the burial of the mutation site (C). The first observation of both the stability and the aggregation analysis is that the observed changes are not discrete but follow a smooth distribution from negative to positive change. Second, there are noticeable differences between SNPs and disease mutations, but they cannot be distinguished by a simple cut-off value on the output, as there is large overlap between the distributions. This is confirmed by the P-values obtained from paired student t-tests, which are 0.96 for the stability distributions, 0.99 for the aggregation distributions, and 0.99 for the burial distributions, respectively. For the stability distributions, we see that disease mutations are generally more destabilising than SNPs, but their distributions overlap largely. A similar analysis has been performed on SwissProt variants using the Site Directed Mutator stability predictor [8], and the distributions of stability differences of disease mutations and neutral variations resemble our findings.

In a first series of properties to test as classifiers, we have investigated 13 properties of the amino acid substitution site that contribute to the assessment of the effect of the mutation using the FoldX algorithm (Table 2). Cut off values were generated that varied between the minimal and maximal values measure for the specific property, and the true and false positive rate, and the Matthews correlation coefficient (MCC) were calculated for each cut-off value. Table 2 lists the data for both the best MCC and the MCC90, i.e. the coefficient that is measured at high specificity (true negative rate = 90%). The corresponding ROC curves for these analyses can be found in Supplementary Figure S2 in Additional file 1.

**Table 2:** Predictive power of structural properties of the modeled variant proteins.

**Property**	**FPR**	**TPR**	**Best MCC**	**Threshold**	**MCC90**
*FoldX energy evaluation*					
Overall stability of residue	14	33	0.22	1.61	0.19
Backbone H bond	32	72	0.40	-1.05	0.22
Sidechain H bond	99	100	0.07	-1.76	*<*0
Electrostatics	86	93	0.11	-0.10	-0.01
Entropy side chain	59	80	0.22	0.32	0.05
Entropy main chain	13	27	0.18	1.96	0.10
Van der Waals contribution	25	47	0.23	-0.98	0.15
Solvation hydrophobic	10	22	0.16	-0.6	0.16
Solvation polar	42	70	0.28	1.5	0.06
Van der Waals clash	18	33	0.17	0.22	0.15
Side chain burial	51	67	0.16	0.43	-0.1
Main chain burial	59	83	0.26	0.73	0.05
*Entropy by sampling of possible side chain conformations*					
Entropy side chain	72	84	0.15	0.93	0

The false positive rate (FPR = 1 - specificity) and the true positive rate (TPR = sensitivity) for the threshold on the specific property that gave the best Matthews correlation coefficient (MCC) are shown. MCC90 is the Matthews correlation coefficient for a specificity of 90% (i.e. 10% false positive rate). The ROC curves corresponding with the evaluation of all properties can be found in Supplementary Figure S2 in Additional file 1. FoldX was used to evaluate both the overall stability contribution of the amino acid substitution site in the modeled structure and the various factors involved in this stability. The entropy of the variant amino acid was calculated using a sampling strategy to assess the possible side chain conformations allowed at the substitution site. Both stability and entropy were calculated for all mutations and for a subset of buried mutations (side chain burial < 0.5) and surface mutations (side chain burial ≥ 0.5). Corresponding ROC curves are shown in Supplementary Figure S3 in Additional file

The same strategy was then applied to predicted values of structural differences between mutant and wild type proteins (24 properties). Statistics were calculated for stability and entropy parameters, as well as for differences concerning protein aggregation, amyloidosis and chaperone binding (Table 3, Supplementary Figure S3 in Additional file 1).

**Table 3:** Predictive power of the differences between wild type and variant proteins for different structural properties.

**Property**	**FPR**	**TPR**	**Best MCC**	**Threshold**	**MCC90**
*FoldX energy evaluation*					
Overall stability difference	73	85	0.15	-0.45	0.14
Overall stability diff. (surface)	0	8	0.2	3.1	0.13
Overall stability diff. (buried)	21	44	0.25	2.64	0.12
Backbone clash	91	99	0.18	-1.00	-0.02
Backbone H bond	59	83	0.26	-0.025	0.06
Sidechain H bond	79	92	0.18	-0.13	-0.14
Electrostatics	6	18	0.18	0.15	0.16
Entropy main chain	6	18	0.18	0.15	0.04
Entropy side chain	64	74	0.11	-0.125	-0.05
Solvation hydrophobic	57	75	0.19	-0.15	-0.03
Solvation polar	22	36	0.15	0.20	-0.05
Torsion clash	1	3	0.07	1.00	-0.05
Van der Waals contribution	7	14	0.11	0.89	0.10
Van der Waals clash	98	100	0.10	-1.60	0.02
*Entropy difference by sampling of possible side chain conformations*					
FoldX entropy difference	85	92	0.11	-1.85	-0.02
FoldX entropy diff. (buried)	96	100	0.14	-2.70	-0.05
FoldX entropy diff. (surface)	37	57	0.20	-0.10	0.02
*Aggregation properties*					
Tango	1	3	0.07	39.9	0
Tango (positive, more aggr.)	14	22	0.10	16.37	0
Tango (negative, less aggr.)	69	78	0.10	-8.00	0
Waltz	0	1	0.07	748.97	0
Waltz (positive, more aggr.)	16	21	0.06	677.15	0
Waltz (negative, less aggr.)	99	100	0.07	-2412.78	0
Limbo	17	33	0.18	5.45	0

FoldX was used to evaluate both the overall stability difference between wild type and variant structure, and the constituting contributions leading to this stability difference. The entropy difference caused by the amino acid substitution was calculated using a sampling strategy to assess the possible side chain conformations allowed at the substitution site. Both stability and entropy difference were calculated for all mutations and for a subset of buried mutations (side chain burial < 0.5) and surface mutations (side chain burial ≥ 0.5). Corresponding ROC curves are shown in Supplementary Figure S2 in Additional file 1.

The results obtained from these detailed analyses are unanimous: none of the parameters evaluated can be used to separate the data. All MCC values are close to zero, and thus the predictions are no better than a random predictor would perform on the data. The high accuracy of FoldX for quantitative stability prediction has been proven in various studies [7,9,10], so we have high confidence in our stability estimations. In accordance with the analyses of Worth and co-workers [8], we find that high stability differences alone are no sufficient criterion to distinguish deleterious mutations and neutral variation. These results show that the dominant effect of for instance stability that was proposed in earlier large-scale studies [5,21] can not always be generalised for other data.

The fact that none of the properties representing conformational differences between wild type and variant protein contain enough information to distinguish neutral and deleterious variation implies that large-scale classification based on singular structural properties is not feasible and requires a better understanding of how the complex interplay between biophysical and biochemical properties of a protein conspire to different tolerance for mutations in different proteins. Although we can predict the structural and functional impact of a mutation of a protein, it is not always feasible to translate this into a prediction of the overall phenotypic effect, i.e. will the mutation result in a disease phenotype or not.

Recent studies that combine structural and evolutionary information using machine learning techniques are able to classify relatively large data sets obtained for the SwissProt database successfully (summarised in Supplementary Table S2). Although the combination of these two types of information improves classification of disease mutations greatly, the incorporation of sequence conservation measures may obscure the mechanism underlying disease. Low frequency substitutions at conserved positions suggest that the mutation will not be tolerated, but will not teach us what the underlying reason of disease is. Although knowing that an amino acid is critical for correct function is of course useful, in a structural bioinformatics approach the focus is more on the molecular mechanism underlying disease.

A simple combination of the SNPeffect structural bioinformatics toolsuite on our evaluation data set showed that in our case, at least a linear combination of these methods is not sufficient to classify the data (TPR = 0.73, TNR = 0.27, MCC = 0). Although we have not fully explored the predictive power of the properties in a more sophisticated approach, such as machine learning techniques that use non-linear combinations (e.g. neural networks, support vector machines), the results obtained in the previous analyses have highlighted a major issue in disease mutation classification. The failure of the classification is mainly due to false positives, i.e. neutral mutations that are predicted to be deleterious. To assess the "predictiveness" of our data set, we applied the well-established evolutionary method SIFT [22] to our data and found that SIFT was also not able to classify effectively the data. In fact the results were even worse than our naive classifier (TPR = 0.69, TNR = 0.21, MCC = -0.12).

As an illustration of the influence of the data set used for evaluation on the performance of a predictor, we list the results for the variation in performance of SNP classification of SIFT, that uses evolutionary information to label SNPs (Supplementary Table S3). The Matthews correlation coefficient varies between -0.12 on our data set over 0.25 on human mutagenesis data, up to 0.59 on the HIV-1 protease mutagenesis set in the original SIFT paper [22]. Although the methodology and underlying data (i.e. the BLOCKS database) is certainly sound, and there is no question that SIFT in most cases can be trusted to evaluate whether or not an amino acid change is tolerated in an evolutionary sense, this variability in classification success illustrates the importance of the choice of training and test data to build and evaluate predictors. SIFT was trained to classify mutations that disrupt the function of a protein, and may suffer from the same limitations as our structural approach. The ability to predict which mutations will affect function does not imply the ability to predict which mutations cause disease.

To date, we have not explored a machine learning technique that incorporates the functional effects predicted by our tools. Some of the problems above may be improved by using non-linear combinations of the structural properties. Since we pose great value in the interpretation of the classification, rule based techniques such as decision trees are our prime choice of machine learning technique. Studies using random forests to classify SNPs show results similar to other state-of-the-art classification techniques as support vector machines [23], and we plan to implement such techniques using the structural parameters described in this manuscript in the near future.

## Conclusion

The concept of using the molecular phenotypic effect of a nsSNP to assess its effect on the structure and function of the protein it alters was first introduced by Bork and co-workers [1]. The question has been raised to how much of this molecular phenotype is necessary to evaluate the contribution of a SNP to a disease phenotype: are there singular dominant properties that determine the impairment of structure and function, or do we need to consider the full ensemble of molecular properties to interpret the impact of the SNP? Other research groups have proposed that single properties such as stability [5] and solvent accessibility [2] can be used to classify SNPs. We have examined all the individual structural bioinformatics tools that were proposed in the SNPeffect toolsuite [24] for their ability to act as a binary classifier for deleterious and neutral SNPs. Neither of the individual properties that were examined could serve this purpose. Because several approaches were able to classify similar data sets as the one we have used, we applied the most used evolutionary method, SIFT [25], to our data set. As it was not able to classify our data set accurately, we argued that generalisation of the results presented by the state of the art classifiers might be an important issue. We illustrated this problem with the variability of performance of SIFT on 8 different data sets used in various analyses.

From these analyses we concluded that strict classification of SNPs is not feasible at the time, both because there are still many technical difficulties to overcome, and because the biological interpretation of the molecular phenotype in relation to a disease phenotype is a complex matter. Even at the single molecule level, we cannot assess how tolerant a specific protein is to structural variation. The inherent rigidity of a protein might influence the change in stability that is allowed before severe conformational changes are introduced. Furthermore, on the cellular level biological interpretation is even harder: we can not predict the role of the protein quality control system plays in this tolerance level, not all interactions are described at the molecular level, and much more. Even if we can predict the molecular effect accurately, this might not necessarily result in a disease phenotype because of functional redundancy of the protein.

However, not being able to classify human variation into disease mutations and neutral or beneficial variation does not mean that this approach or the methods developed are useless. By using high quality bioinformatics tools, we can select from a large pool of variations the candidates that are interesting for detailed investigation. This in itself is a valuable contribution, because the amount of variation data available is too massive to be investigated experimentally. *In silico *analyses can and will be used successfully as an addition to *in vitro *and *in vivo *studies.

## Methods

### Assembly of data sets

Statistics on the structural coverage and validation status of human non synonymous coding SNPs were performed on data from the Ensembl human variation database release 44, containing 12.2 million SNPs, of which 133698 cause an amino acid variation in a known transcript. The mapping of SNPs on protein structures was evaluated using the "ensppdbmapping" DAS service provided by the SPICE server [26]. Positive and negative data sets (disease related mutations and polymorphisms) for the evaluation of SNP classification were designed with data from the SwissProt variation index [27] in the UniProt knowledge base (version 52.0, March 2007, [28]) that were mapped onto known PDB structures and high quality homologs thereof. The quality criteria described in the results section (models with resolution of 2.5 Å or higher, sequence identity of 80% or more) lead to structural models of 400 SNPs (negative) and 240 disease associated mutations (positive).

### Structural bioinformatics tools

We have used the FoldX force field [29] for all mutant properties regarding structural location, protein stability and its various components, the Tango [30] and Waltz [[31], submitted] algorithms to assess the propensity for aggregation of wild type and variant proteins, and the Limbo algorithm [[17], submitted] to evaluate the chaperone-binding properties of amino acid sequences. A novel tool developed by Lenaerts *et al *(unpublished) was used to estimate the entropy of a specific amino acid site in a high-resolution structure.

#### FoldX

The FoldX force field was developed for the fast and accurate estimation of the free change upon mutation on the stability of a protein or a protein complex [9,14,29,32]. It uses an all-atom representation of these macromolecules, and has been validated on a test database of more than 1000 mutants from more than 20 different proteins. It currently yields a correlation of 0.78 with a standard deviation of 0.41 kcal/mol.

Modelling and evaluation of mutations in FoldX is performed with the *BuildModel *command. It is used first to model a homologous sequence on a structural model and to optimise the side chains to fit the new sequence, and then to evaluate the effect of a single amino acid variation. The Gibbs free energy of a protein is calculated with the Stability command. The various structural parameters used in the classification tests (backbone clash, backbone H bond formation, sidechain H bond formation, electrostatics, solvation of hydrophobic residues, solvation of polar residues, torsion clash, Van der Waals contribution, Van der Waals clash)

#### Entropy calculations based on side chain sampling

In addition to the entropy calculations intrinsic to the FoldX force field, we use a novel method based on extensive sampling of side chain conformations as developed by Lenaerts *et al. *(unpublished). The sampling method produces for each side chain the probability (*P*(*X*)) of finding the residue's side chain in a particular conformational state. From these probabilities entropy can easily be derived:



The method uses a rotamer database based on conditional statistics of dihedral angles derived from the WHAT IF data set [33]. All amino acids from this data and their corresponding dihedral angles (10° bin) were used to derive the following probabilities: *P*(*χ*_*i*_), *P*(*χ*_*i*_|*χ*_*i*-1_) and *P*(*χ*_*i*_|*χ*_*i*-1_, *χ*_*i*-2_), except for *χ*_1_(*P*(*χ*_1_) and *P*(*χ*_1_|*ϕ*, *ψ*)). A set of *n *random rotamers can be derived from the probability distribution thus calculated. This will allow sampling of rotamers with greater resolution than classical rotamer libraries.

The sampling itself is performed by Monte Carlo based sampling method with Metropolis criterion (at 298 K). The Metropolis criterion states that a certain conformational change is accepted with a probability *p *that depends on the free energy change ΔΔ*G *associated with the conformational change as given by the following formula:



The free energy of each change is determined with FoldX.

#### Tango

The *β*-aggregation prediction algorithm Tango [30] uses a statistical mechanics approach to represent a competition between major conformational states: the random coil and the native conformations, as well as *β*-turn, *α*-helix and *β*-aggregate. Two windows of variable length slide over the sequence, and each such window can populate these conformational states according to a Boltzmann distribution. The frequency of population of each structural state for a given segment will be relative to its energy, which is derived from statistical and empirical parameters. To predict the *β*-aggregating segments of a peptide, Tango calculates the partition function of the phase space involving these conformational states. In our analysis we have used Tango to calculate the difference in aggregation tendency that results from an single amino acid variation.

#### Waltz

Current methods for the prediction of the sequence determinants of amyloidosis suffer from two major problems: overpredicting amorphous cross *β *aggregates and missing amylogenic sequences that are enriched in the polar Q and N residues, such as the prion protein. The Waltz algorithm [[31], submitted] tackles these problems by taking into account amyloid hexapeptides from 48 new amyloid forming sequences, derived from 31 proteins. About half the proteins in this extended data set were not previously known to contain amyloidogenic sequences such as presenilin-2, titin and myosin. Waltz combines terms from amino acid sequence scoring in the learning set, physical property analysis and homology modelling. The method shows 84% sensitivity at 92% specificity on the AmylHex data set [34], and correctly identifies mutations in human proteins known to be associated with amyloid deposition.

#### Limbo

Limbo is a Hsp70 binding site predictor that was built using a dual method combining sequence and structural information [[17], submitted]. Experimental DnaK binding data of 53 non-redundant peptide sequences was used to generate a sequence-based position-specific scoring matrix (PSSM) based on logarithm of the odds scores. Following an *in silico *alanine scan of the substrate peptide in the crystal structure of a DnaK-substrate complex (PDBID 1DKX) using FoldX, a structure-based PSSM that reflects the individual contribution of certain substrate residue types for DnaK binding was generated. The Limbo DnaK binding site predictor was obtained by combining the structure-based PSSM with a normalisation factor of 0.2 with the sequence-based PSSM. Limbo is able to correctly predict 89% of the true positives in a tested peptide set (high sensitivity), with a concurrent amount of only 5.9% false positives for a specific score threshold (high specificity). The robustness of the predictor was evaluated with a cross-validation test, resulting in a true positive rate of 72% true positives and a false positive rate of 5.9%. The predictor was able to identify an entire known DnaK binding site in the heat-shock promoter *σ *32 [35]. We have used Limbo to rank mutated proteins according to their DnaK binding affinity.

### Evaluation of classification

Several statistics can be calculated to assess the accuracy of a binary classification. We have used the true positive rate or sensitivity (*TPR*), true negative rate or specificity (*TNR*), false positive rate (*FPR*) and false negative rate (*FNR*) can be calculated using the true positives (*TP*), true negatives (*TN*), false positives (*FP*) and false negatives (*FN*) as follows:



The Matthews Correlation Coefficient [36] is used in machine learning as a measure of the quality of binary classifications, and can be calculated as follows:



Other measures, such as the proportion of correct predictions, are not useful when the two classes are of very different sizes. For example, assigning every object to the larger set achieves a high proportion of correct predictions, but is not generally a useful classification.

The MCC is generally regarded as a balanced measure which can be used even if the positive and negative classes are of very different sizes. It returns a value between -1 and +1. A coefficient of +1 represents a perfect prediction, 0 an average random prediction and -1 the worst possible prediction.

In a Receiver Operating Characteristic (ROC) curve the true positive rate (TPR) is plotted in function of the false positive rate (FPR) for different cut-off points. Each point on the ROC plot represents a sensitivity/specificity pair corresponding to a particular decision threshold. A test with perfect discrimination between positives and negatives has a ROC plot that passes through the upper left corner (100% sensitivity, 100% specificity). Therefore the closer the ROC plot is to the upper left corner, the higher the overall accuracy of the test [37].

## Competing interests

The authors declare that they have no competing interests.

## Authors' contributions

Conceived and designed the experiments: JR JS FR. Performed the experiments: JR. Analysed the data: JR JS FR. Wrote the paper: JR.

## Supplementary Material

Additional file 1 Several of the less critical figures and tables are added as supplementary material, together with detailed descriptions of the structural bioinformatics tools used. Click here for file
