# Supplementary material for: Using structural bioinformatics to investigate the impact of non synonymous SNPs and disease mutations: scope and limitations
Source: BMC Bioinformatics. 2009 Aug 27;10(Suppl 8):S9. doi: 10.1186/1471-2105-10-S8-S9 (PMC2745591; doi:10.1186/1471-2105-10-S8-S9)
Supplement: Additional file 1 — Several of the less critical figures and tables are added as supplementary material, together with detailed descriptions of the structural bioinformatics tools used. [file 1471-2105-10-S8-S9-S1.pdf]

Using structural bioinformatics to investigate the impact of non synonymous SNPs and disease mutations: scope and limitations
Supplementary Material

Joke Reumers[1], Joost Schymkowitz[1] and Fréderic Rousseau*[1]

[1]Switch Laboratory, VIB, Vrije Universiteit Brussel, Pleinlaan 2, 1050 Brussels, Belgium

Email: Joke Reumers - joke.reumers@vub.ac.be; Joost Schymkowitz - joost.schymkowitz@vub.ac.be; Fréderic Rousseau*- frederic.rousseau@vub.ac.be;

*Corresponding author

Tables

Supplementary Table S1 - Types of data sets used to train and test SNP classifiers.
The column "Size of data set" refers to the range of the data sets used, i.e. the smallest and largest data sets.

**Origin data set**	**Size of data set**	**Number of studies**	**References**
Neutral variations			
Mutagenesis studies	111-3706	9	[1–9]
Orthologs	888-16682	3	[3, 9, 10]
SwissProt SNP	502-12944	6	[3, 8, 11–14]
OMIM	558	1	[15]
dbSNP	5177-21471	2	[16, 17]
Disease mutations			
Mutagenesis studies	159-1750	8	[1–9]
COSMIC database	879	1	[18]
HGMD	3768-10263	1	[9]
OMIM	879-2249	5	[3, 8, 13, 15, 18]
SwissProt Disease	175-9610	9	[3, 8, 10–14, 19, 20]
Data from Haluschka *et al.* [21]	209	1	[20]
Data from Cargill *et al.* [22]	185	2	[19, 20]

Supplementary Table S2 - Performance of state-of-the-art predictors on representative data sets. The performance of a few selected tools on SwissProt disease associated mutations and SNP data are shown. The false positive rate (FPR = 1 - specificity), the true positive rate (TPR = sensitivity) and the Matthews correlation coefficient (MCC) are shown where available. Although all analyses use the variation data from the SwissProt knowledge base, the effective size of the data set varies between analyses.

**Study**	**Method**	**FPR**	**TPR**	**MCC**	**Size data set**
Bao *et al* [11]	Random Forest	0.3	0.76	0.46	205
Capriotti *et al* [13]	HybridMeth	-	-	0.46	21185
Karchin *et al* [14]	SVM	0.2	0.81	0.61	3691
Ng & Henikoff [19]	SIFT	0.19	0.69	0.50	5333
Wang & Moult [20]	Stability	0.3	0.9	0.61	262
Worth *et al* [16]	Combined	0.09	0.32	0.28	9143
Yue & Moult [9]	SVM	0.15	0.74	0.59	6077

Supplementary Table S3 - Variation of the performance of SIFT on different data sets. The false positive rate (FPR = 1 - specificity), the true positive rate (TPR = sensitivity) and the Matthews correlation coefficient (MCC) are shown where available.

**Study**	**Dataset**	**FPR**	**TPR**	**MCC**
Bao *et al* [11]	Test set	0.33	0.62	0.29
Saunders *et al* [8]	Human	0.4	0.65	0.25
Ng & Henikoff [7]	lac I repressor	0.22	0.57	0.36
Ng & Henikoff [7]	HIV 1-protease	0.3	0.88	0.59
Ng & Henikoff [7]	T4 lysozyme	0.41	0.72	0.31
Ng & Henikoff [19]	SwissProt disease	0.19	0.69	0.50
Worth *et al* [16]	SwissProt + dbSNP	0.41	0.71	0.30
Our evaluation	SwissProt	0.79	0.69	-0.12

Figures

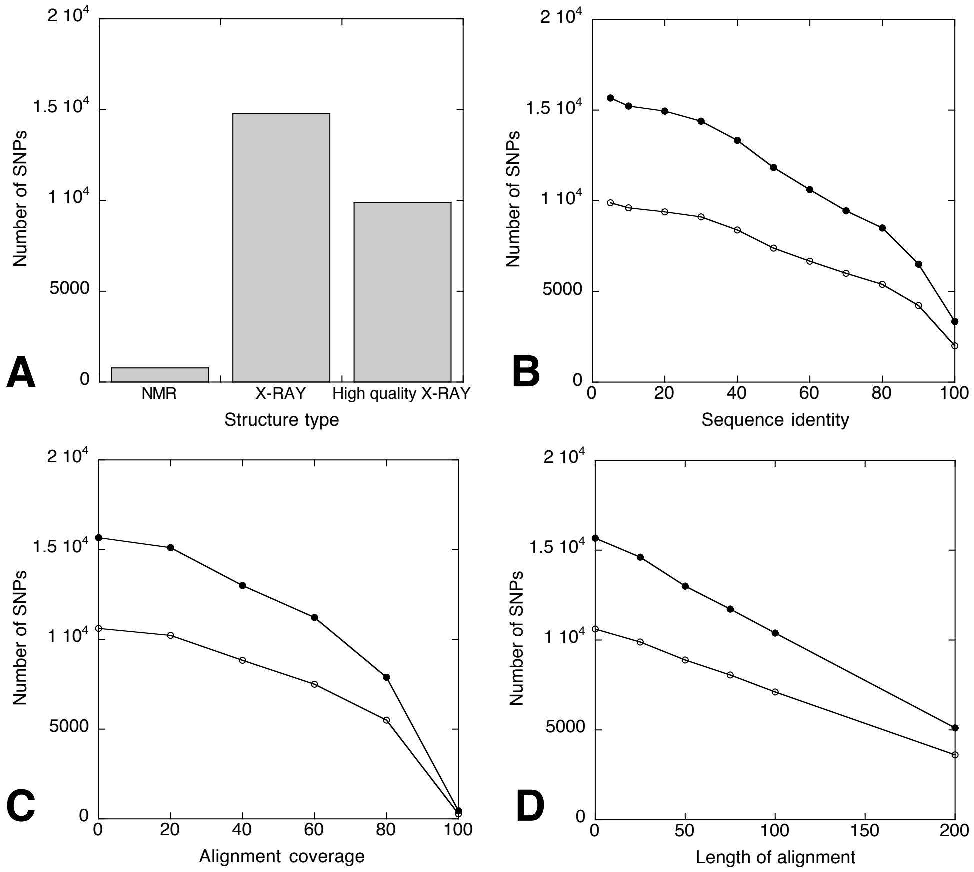

Figure S1. Structural coverage of Ensembl non synonymous SNP data. A. Number of SNPs in structures determined by NMR and X-ray crystallography studies or models of these structures. 11% of all non synonymous SNPs can be mapped on crystallography structures, and 7% of all SNPs can be modeled on a high-quality X-ray structure (resolution ≤ 2.5Å). **B. Number of SNPs covered by structural data versus the sequence identity between the query sequence and the structural model.** The number of SNPs that can be modeled on X-ray structures (•) decreases from 15% of all nsSNPs (15685 nsSNPs, 5% sequence identity) to 2.5% (3341) of all SNPs for which the structure of the wild type sequence has been determined experimentally (100% sequence identity). When only high quality structures are considered (○), this amount is reduced by half to 7.4% for a sequence identity of 5% and 1.5% for exact models. **C. Number of SNPs covered by structural data versus the sequence coverage of the wild type sequence.** There are almost no SNPs for which the full length of the protein sequence is covered (100% coverage), but for 80% coverage almost 8000 SNPs can be selected, of which circa 5500 in high quality structures. **D. Number of SNPs covered by structural data versus the length of the alignment between protein sequence and structural model.** About a third of the SNPs that can be modeled are located in a structural alignment that is less than 100 amino acids long, both for models based on all X-ray structures (•) and based on high resolution structures only (○).

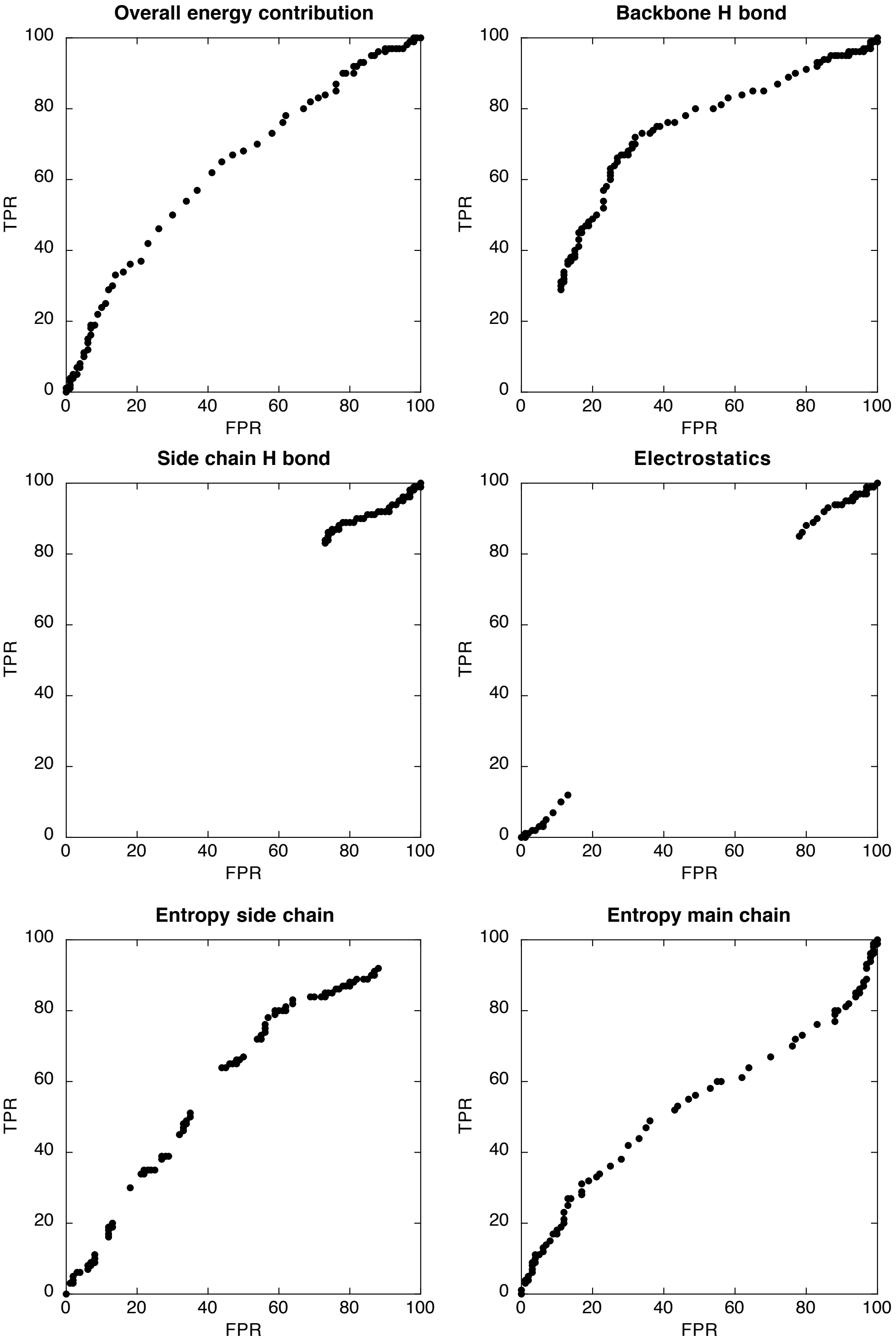

Figure S2. ROC curves for classification of disease mutations and neutral variation by using structural properties of the amino acid substitution site.

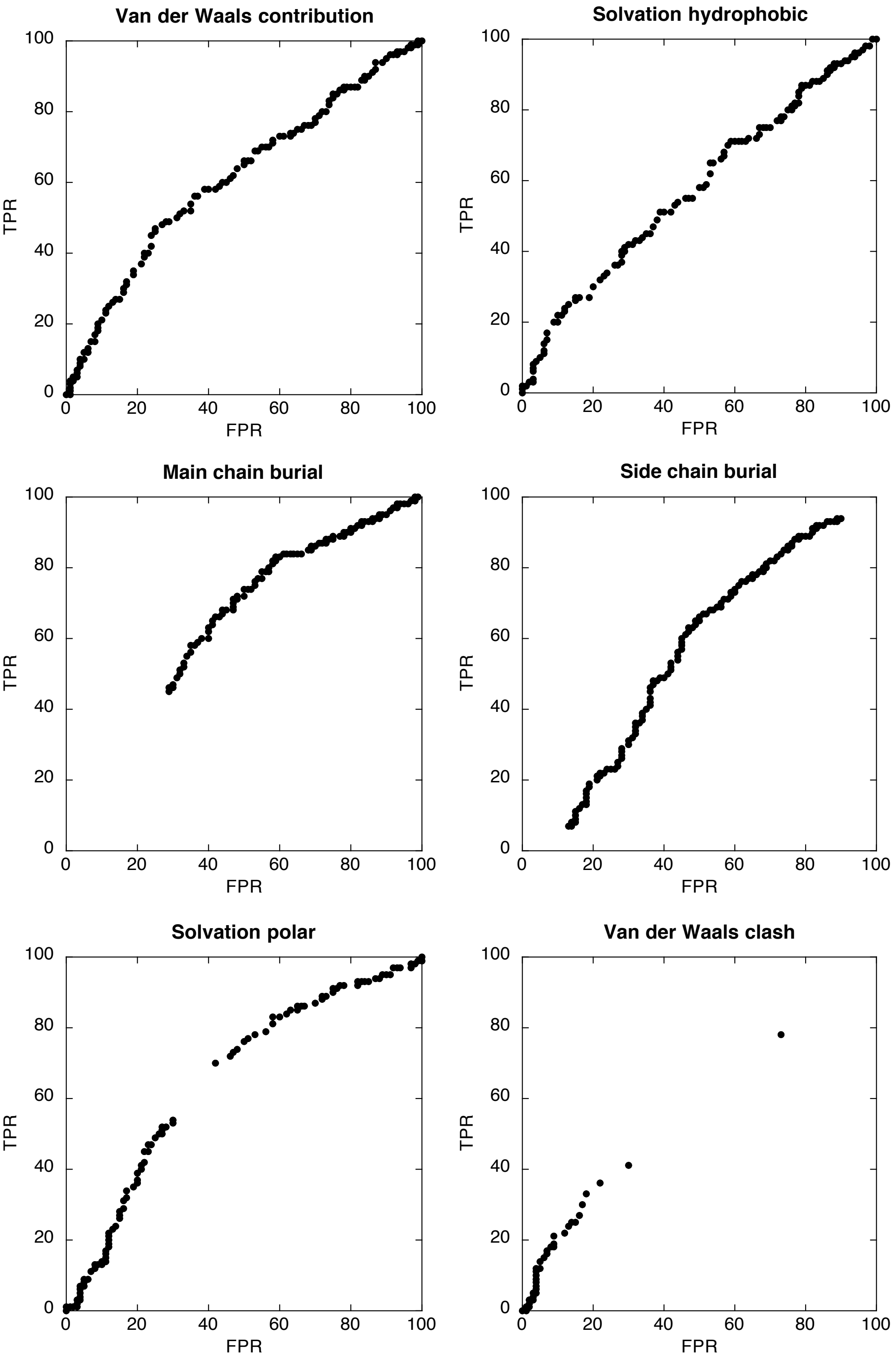

Figure S2 ***(continued).*** ROC curves for classification of disease mutations and neutral variation by using structural properties of the amino acid substitution site.

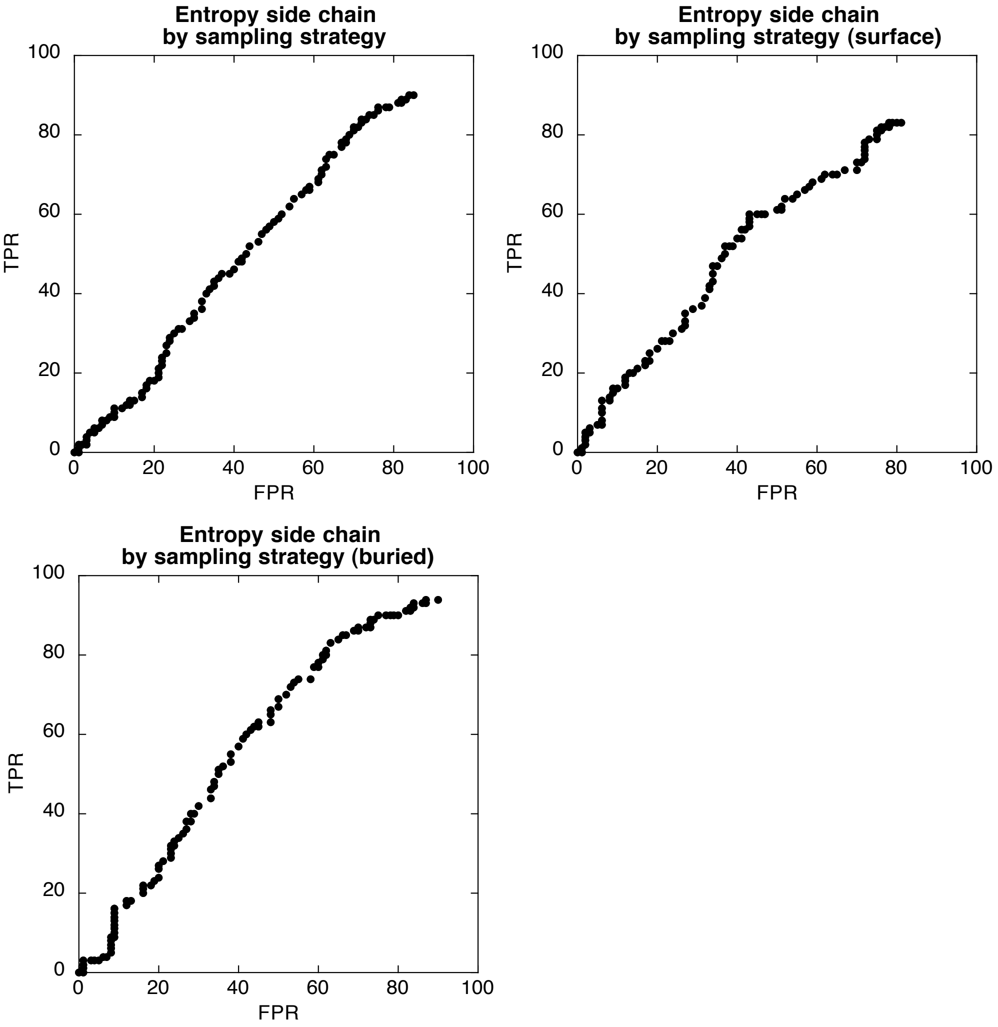

Figure S2 ***(continued).*** ROC curves for classification of disease mutations and neutral variation by using structural properties of the amino acid substitution site.

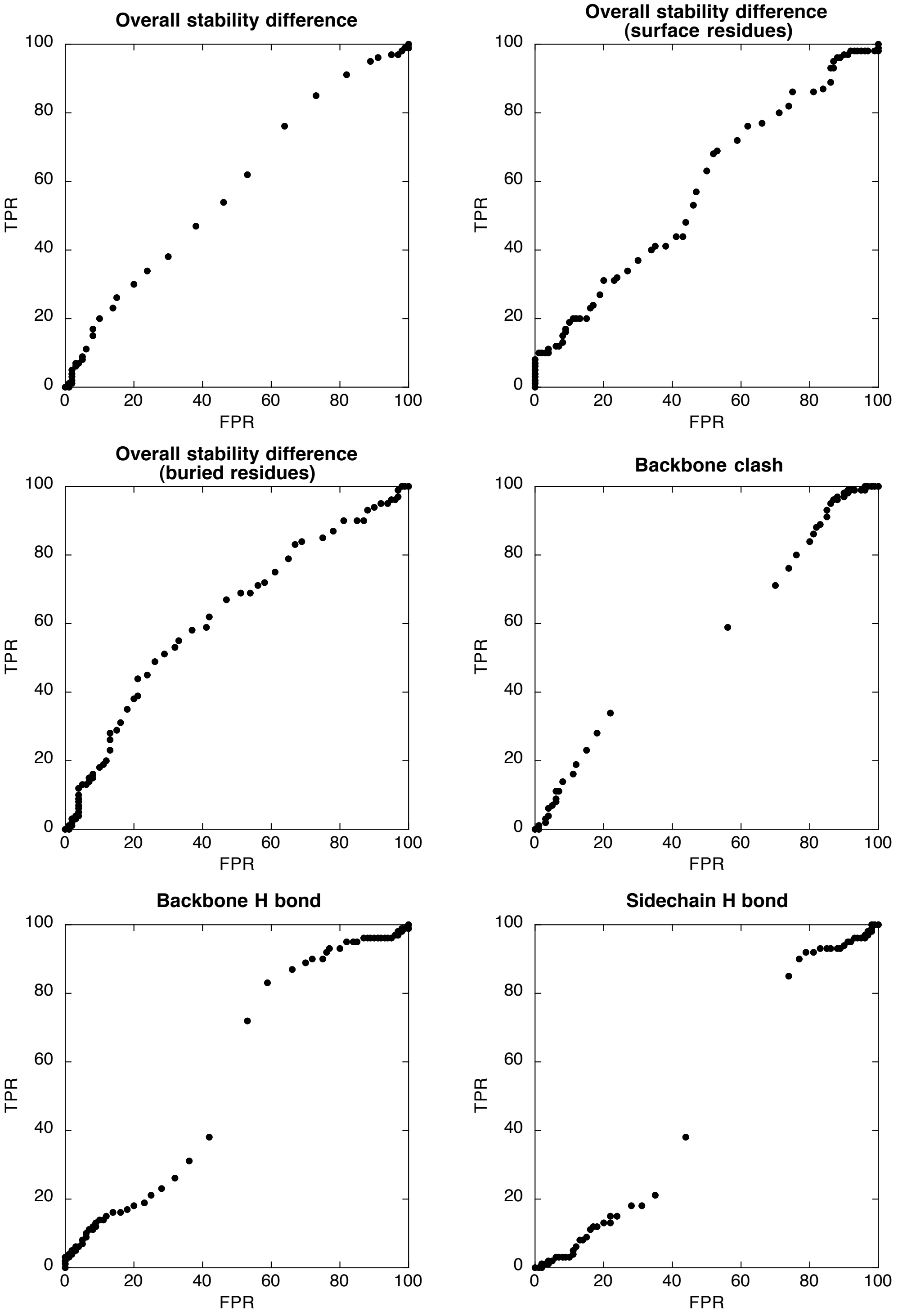

Figure S3. ROC curves for classification of disease mutations and neutral variation by using structural differences between the wild type and variant protein.

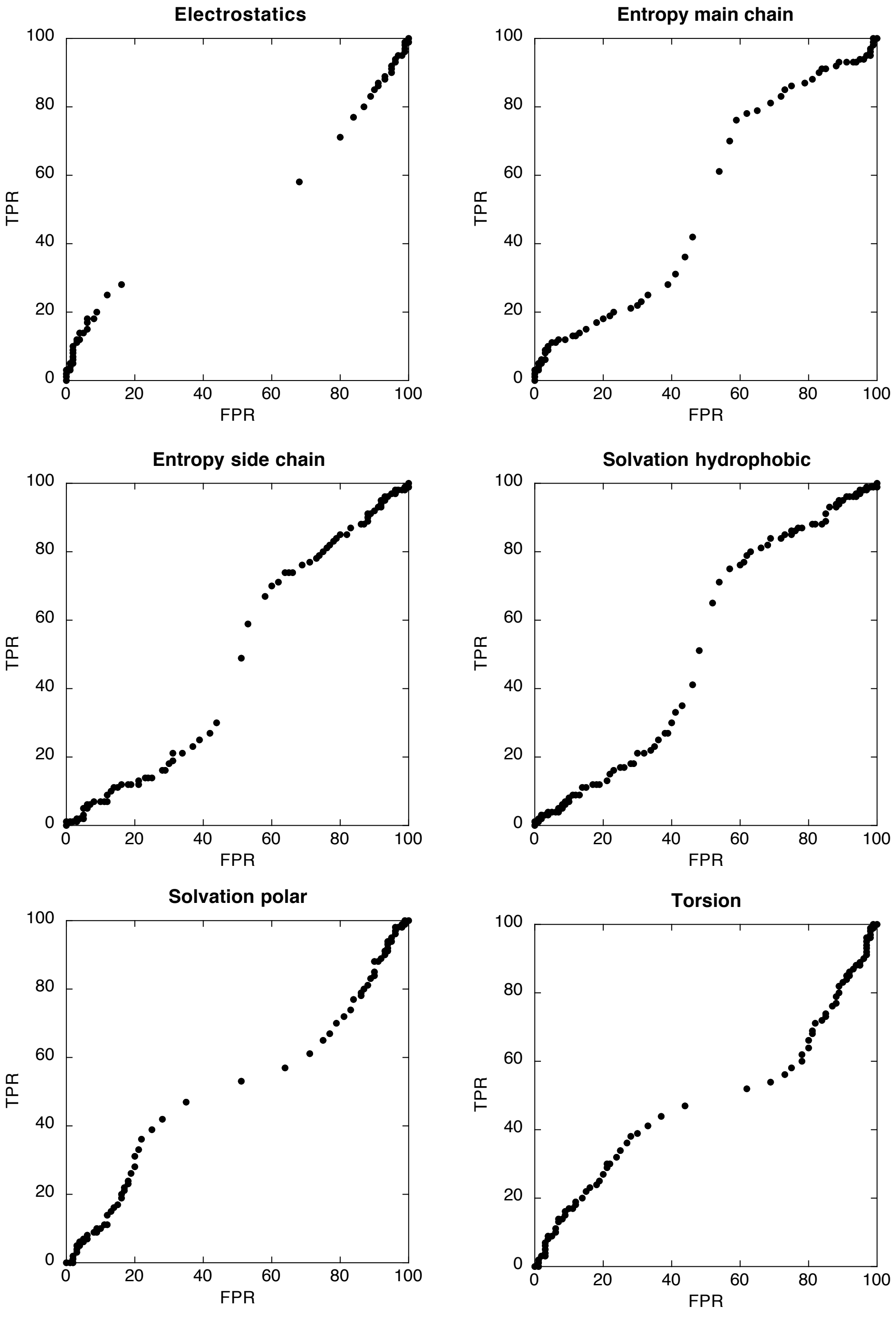

Figure S3 ***(continued).*** ROC curves for classification of disease mutations and neutral variation by using structural differences between the wild type and variant protein.

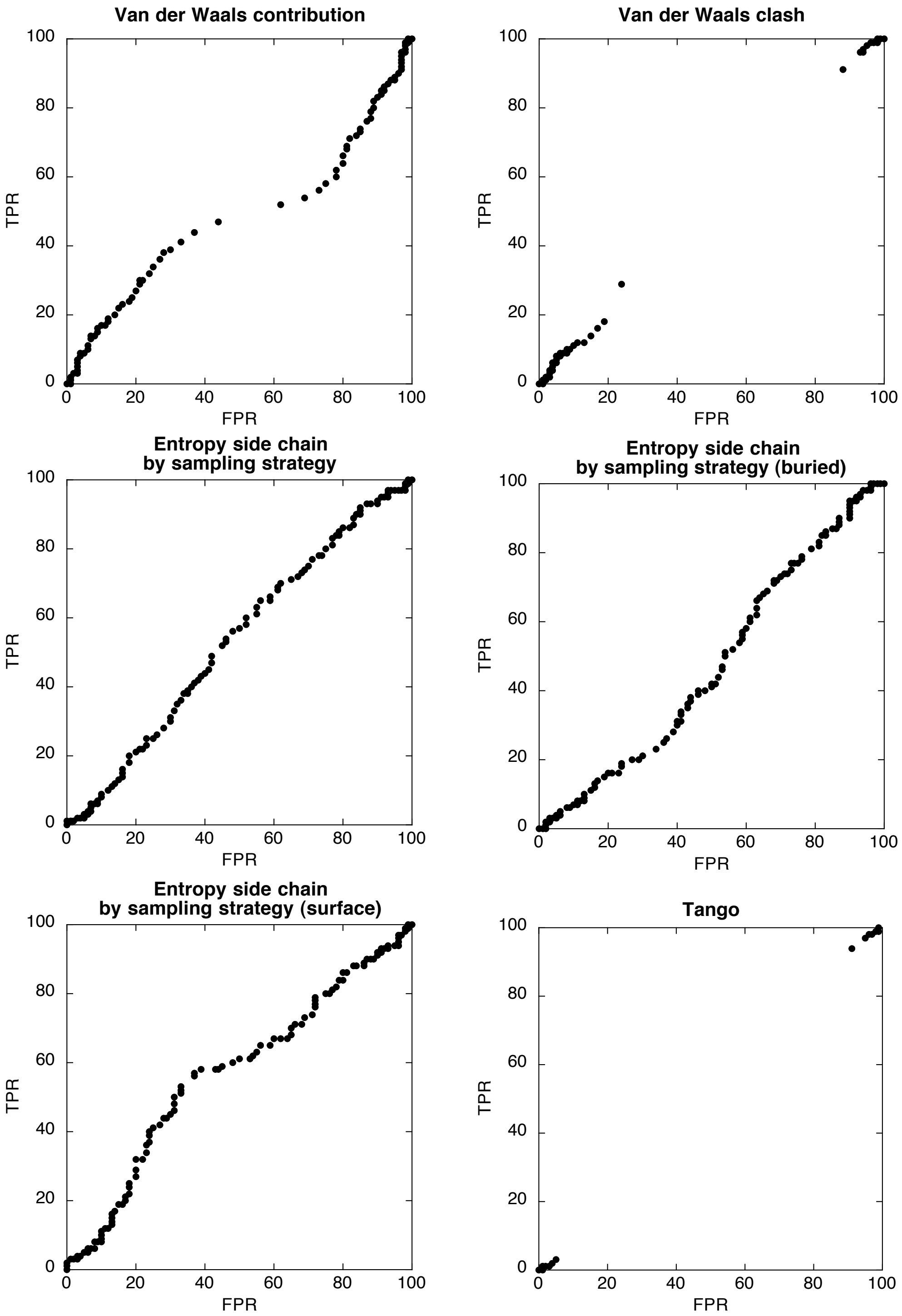

Figure S3 ***(continued).*** ROC curves for classification of disease mutations and neutral variation by using structural differences between the wild type and variant protein.

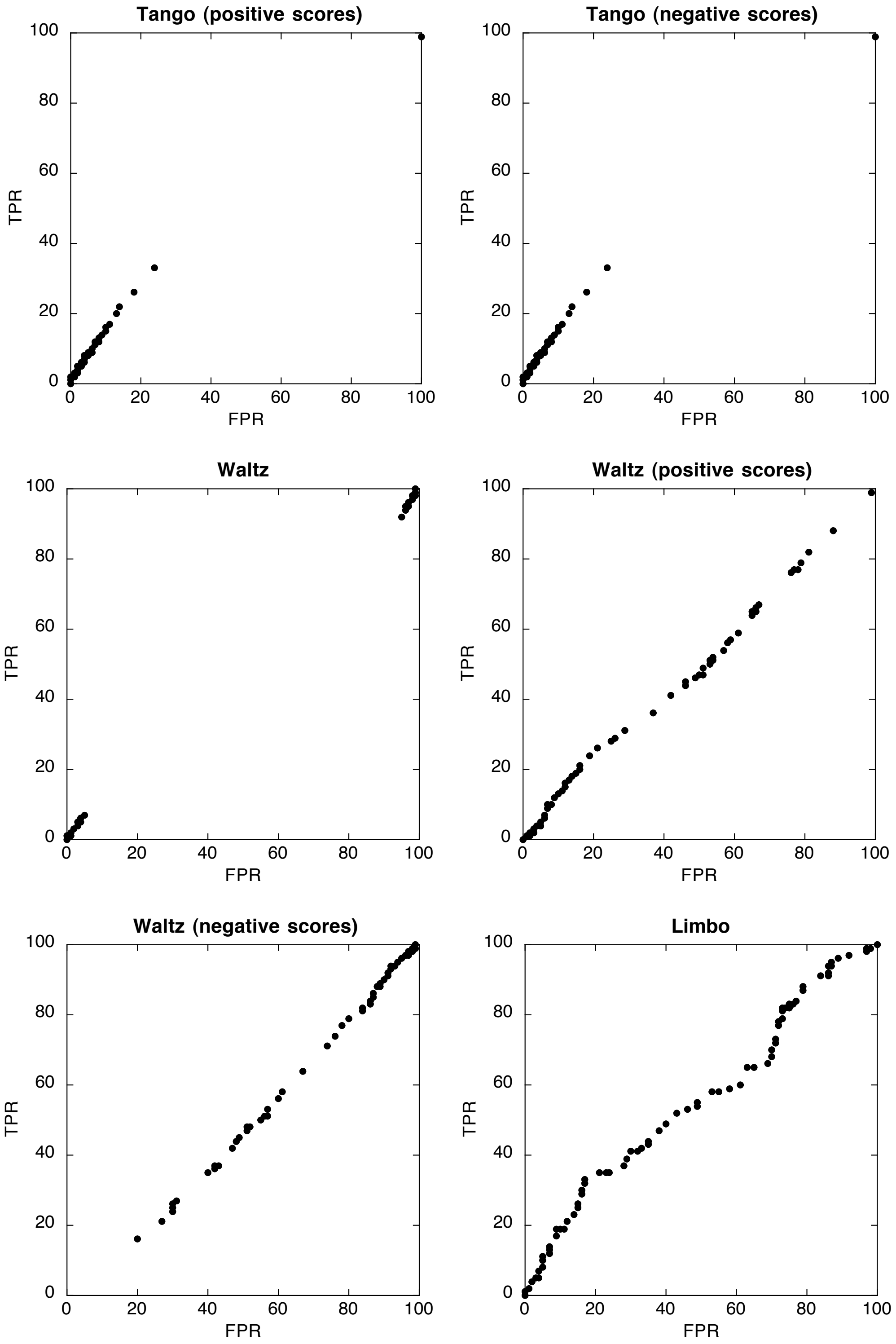

Figure S3 ***(continued).*** ROC curves for classification of disease mutations and neutral variation by using structural differences between the wild type and variant protein.

References

1. Chasman D, Adams RM: **Predicting the functional consequences of non-synonymous single nucleotide polymorphisms: Structure-based assessment of amino acid variation**. *J Mol Biol* 2001, **307**(2):683–706.
2. Clifford RJ, Edmonson MN, Nguyen C, Buetow KH: **Large-scale analysis of non-synonymous coding region single nucleotide polymorphisms**. *Bioinformatics* 2004, **20**(7):1006–1014.
3. Ferrer-Costa C, Orozco M, de la Cruz X: **Sequence-based prediction of pathological mutations.** *Proteins* 2004, **57**(4):811–819.
4. Jiang R, Yang H, Sun F, Chen T: **Searching for interpretable rules for disease mutations: a simulated annealing bump hunting strategy.** *BMC Bioinformatics* 2006, **7**:417.
5. Krishnan VG, Westhead DR: **A comparative study of machine-learning methods to predict the effects of single nucleotide polymorphisms on protein function**. *Bioinformatics* 2003, **19**(17):2199–2209.
6. Needham CJ, Bradford JR, Bulpitt AJ, Care MA, Westhead DR: **Predicting the effect of missense mutations on protein function: analysis with Bayesian networks.** *BMC Bioinformatics* 2006, **7**:405.
7. Ng PC, Henikoff S: **Predicting deleterious amino acid substitutions**. *Genome Res* 2001, **11**(5):863–874.
8. Saunders CT, Baker D: **Evaluation of structural and evolutionary contributions to deleterious mutation prediction**. *J Mol Biol* 2002, **322**(4):891–901.
9. Yue P, Li Z, Moult J: **Loss of protein structure stability as a major causative factor in monogenic disease.** *J Mol Biol* 2005, **353**(2):459–473.
10. Ferrer-Costa C, Orozco M, de la Cruz X: **Characterization of disease-associated single amino acid polymorphisms in terms of sequence and structure properties**. *J Mol Biol* 2002, **315**(4):771–786.
11. Bao L, Cui Y: **Prediction of the phenotypic effects of non-synonymous single nucleotide polymorphisms using structural and evolutionary information**. *Bioinformatics* 2005, **21**(10):2185–2190.
12. Bao L, Cui Y: **Functional impacts of non-synonymous single nucleotide polymorphisms: Selective constraint and structural environments**. *FEBS Lett* 2006, **580**(5):1231–4.
13. Capriotti E, Calabrese R, Casadio R: **Predicting the insurgence of human genetic diseases associated to single point protein mutations with support vector machines and evolutionary information.** *Bioinformatics* 2006, **22**(22):2729–2734.
14. Karchin R, Diekhans M, Kelly L, Thomas DJ, Pieper U, Eswar N, Haussler D, Sali A: **LS-SNP: large-scale annotation of coding non-synonymous SNPs based on multiple information sources**. *Bioinformatics* 2005, **21**(12):2814–2820.
15. Stitziel NO, Tseng YY, Pervouchine D, Goddeau D, Kasif S, Liang J: **Structural location of disease-associated single-nucleotide polymorphisms**. *J Mol Biol* 2003, **327**(5):1021–1030.
16. Worth CL, Bickerton GRJ, Schreyer A, Forman JR, Cheng TMK, Lee S, Gong S, Burke DF, Blundell TL: **A structural bioinformatics approach to the analysis of nonsynonymous single nucleotide polymorphisms (nsSNPs) and their relation to disease.** *J Bioinform Comput Biol* 2007, **5**(6):1297–1318.
17. Burke DF, Worth CL, Priego EM, Cheng T, Smink LJ, Todd JA, Blundell TL: **Genome bioinformatic analysis of nonsynonymous SNPs.** *BMC Bioinformatics* 2007, **8**:301.
18. Worth CL, Burke DF, Blundell TL: **Estimating the effects of single nucleotide polymorphisms on protein structure: how good are we at identifying likely disease associated mutations?** In *Proceedings of Molecular Interactions - Bringing Chemistry to Life* 2006.
19. Ng PC, Henikoff S: **Accounting for human polymorphisms predicted to affect protein function**. *Genome Res* 2002, **12**(3):436–446.
20. Wang Z, Moult J: **SNPs, protein structure, and disease**. *Hum Mutat* 2001, **17**(4):263–270.
21. Halushka MK, Fan JB, Bentley K, Hsie L, Shen N, Weder A, Cooper R, Lipshutz R, Chakravarti A: **Patterns of single-nucleotide polymorphisms in candidate genes for blood-pressure homeostasis.** *Nat Genet* 1999, **22**(3):239–247.
22. Cargill M, Altshuler D, Ireland J, Sklar P, Ardlie K, Patil N, Shaw N, Lane CR, Lim EP, Kalyanaraman N, Nemesh J, Ziaugra L, Friedland L, Rolfe A, Warrington J, Lipshutz R, Daley GQ, Lander ES: **Characterization of single-nucleotide polymorphisms in coding regions of human genes (vol 22, pg 231, 1999)**. *Nat Genet* 1999, **23**(3):373–373.
